# Chromogranin A Regulation of Obesity and Peripheral Insulin Sensitivity

**DOI:** 10.3389/fendo.2017.00020

**Published:** 2017-02-08

**Authors:** Gautam K. Bandyopadhyay, Sushil K. Mahata

**Affiliations:** ^1^Department of Medicine, University of California San Diego, La Jolla, CA, USA; ^2^Department of Medicine, Metabolic Physiology and Ultrastructural Biology Laboratory, VA San Diego Healthcare System, San Diego, CA, USA

**Keywords:** obesity, insulin resistance, inflammation, chromogranin A knockout, pancreastatin, catestatin

## Abstract

Chromogranin A (CgA) is a prohormone and granulogenic factor in endocrine and neuroendocrine tissues, as well as in neurons, and has a regulated secretory pathway. The intracellular functions of CgA include the initiation and regulation of dense-core granule biogenesis and sequestration of hormones in neuroendocrine cells. This protein is co-stored and co-released with secreted hormones. The extracellular functions of CgA include the generation of bioactive peptides, such as pancreastatin (PST), vasostatin, WE14, catestatin (CST), and serpinin. CgA knockout mice (*Chga*-KO) display: (i) hypertension with increased plasma catecholamines, (ii) obesity, (iii) improved hepatic insulin sensitivity, and (iv) muscle insulin resistance. These findings suggest that individual CgA-derived peptides may regulate different physiological functions. Indeed, additional studies have revealed that the pro-inflammatory PST influences insulin sensitivity and glucose tolerance, whereas CST alleviates adiposity and hypertension. This review will focus on the different metabolic roles of PST and CST peptides in insulin-sensitive and insulin-resistant models, and their potential use as therapeutic targets.

## Introduction

**Figure 1:**
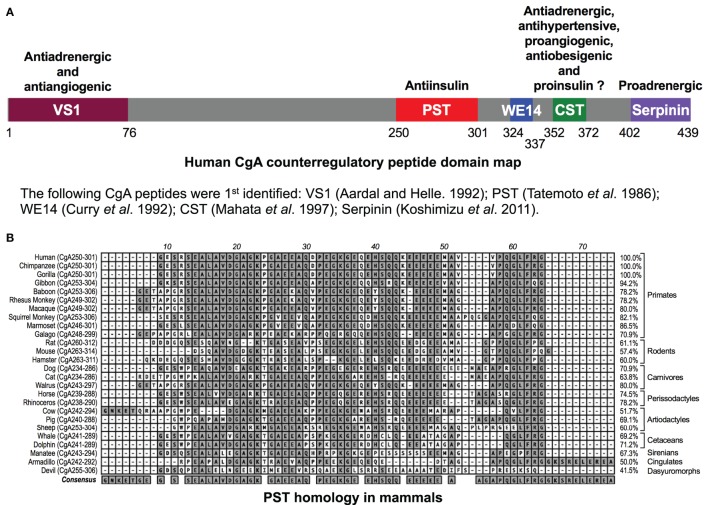
**(A)** Schematic depiction of the domains of the chromogranin A (CgA) protein. Relative locations of vasostatin (VS1), pancreastatin (PST), WE14, catestatin (CST), and serpinin domains in CgA have been illustrated along with the description of their basic functional properties. **(B)** PST homology in mammals. Clustal-W program of MacVector (version 9.0) was used for PST domain alignments across 26 mammalian species. PST amino acid domains were shown on the left and percentage homology as compared to human sequence (100%) was shown on the right. The following gene accession numbers were used for this analysis: human (J03483), chimpanzee (XM_510135), western lowland gorilla (XM_004055595), northern white-cheeked gibbon (XM_003260903), olive baboon (NC_018155.1), rhesus monkey (XM_001092629), crab-eating macaque (AB_169793), Bolivian squirrel monkey (XM_003939842), white-tufted-ear marmoset (XM_002754214), small-eared galago (XM_003786997), Norway rat (XM_346781), house mouse (NM_007693), Chinese hamster (NW_003614307), dog (XM_003639191), cat (XM_003987967), Pacific walrus (XM_004394490), horse (NM_001081814), southern white rhinoceros (XM_004434217), cow (NM_181005), pig (XM_001925714), sheep (XM_004017959), killer whale (XM_004262352), bottle-nosed dolphin (XM_004315772), Florida manatee (XM_004376681), nine-banded armadillo (XM_004475519), and Tasmanian devil (XM_003756143). -, gaps in the alignment.

The human chromogranin A (gene, *CHGA*; protein, CgA) gene encodes a 439-amino-acid mature protein of approximately 48–52 kDa with a coiled-coil structure (1–6). Initially detected in chromaffin granules of the adrenal medulla, this evolutionarily conserved protein is ubiquitously distributed in secretory vesicles of endocrine, neuroendocrine, and neuronal cells. CgA plays a pivotal role in the initiation and regulation of dense-core secretory granule biogenesis and hormone sequestration at the *trans*-Golgi network in neuroendocrine cells (4, 7–9). Increased levels of CgA have been identified in the blood of patients suffering from carcinoids or other neuroendocrine tumors (10–14), heart failure, renal failure, hypertension, rheumatoid arthritis, and inflammatory bowel disease (15–23), indicating an important role of CgA to influence human health and disease (24). Structurally, CgA has 8–10 dibasic sites and is proteolytically cleaved by prohormone convertases (25–27), cathepsin L (28), plasmin (29, 30), and kallikrein (31), generating biologically active peptides including the dysglycemic peptide pancreastatin (PST) (CgA_250–301_) (32, 33); WE14 (hCgA_324–337_) which acts as the antigen for highly diabetogenic CD4^+^ T cell clones (34–38); the vasodilating, antiadrenergic, and antiangiogenic peptide vasostatin 1 (CgA_1–76_) (39–43); the antiadrenergic, antihypertensive, antibacterial, proangiogenic, and antiobesigenic peptide catestatin (CST) (CgA_352–372_) (44–56); and the proadrenergic peptide serpinin (CgA_402–439_) (57, 58). Several of these CgA-derived peptides have opposing counter-regulatory effects. For example, cardiac contractility in rodents is controlled by vasostatin (hCgA_1–76_) and CST (hCgA_352–372_), which are antiadrenergic (51, 59) as well as serpinin (hCgA_402–439_), which is proadrenergic (58) (Figure 1A). Likewise, angiogenesis is controlled by vasostatin acting in an antiangiogenic manner (43, 56) and CST acting as in a proangiogenic manner (50, 56). These CgA-derived peptides, with diverse functions, emphasize the importance of the CgA proprotein in the regulation of physiological functions (Figure 1A). Accordingly, *Chga* whole-body knockout mice present a complex set of metabolic phenotypes and are obese, hyperadrenergic, and hypertensive (48, 60–63). *Chga*-KO mice have become an important model to study the roles of individual CgA-derived peptides through analysis of phenotypes after supplementation (48, 55, 60, 61, 64). Here, we will focus on how two of these peptides, PST and CST, act as important modulators of insulin sensitivity and glucose metabolism.

## PST Inhibits Glucose-Stimulated Insulin Secretion (GSIS)

**Figure 2:**
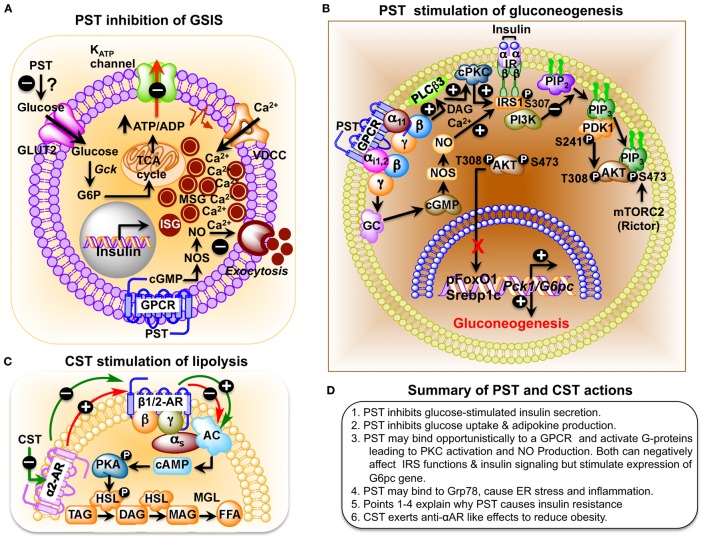
**(A)** Schematic representation of the role of pancreastatin (PST) in the regulation of insulin secretion from pancreatic beta cells. PST-induced nitric oxide (NO) production, following a guanylate cyclase–cGMP–NOS pathway, inhibits glucose-stimulated insulin secretion (GSIS). **(B)** Schematic diagram showing PST inhibition of gluconeogenesis in hepatocytes. PST initiates a GTP-binding protein linked signaling cascade leading to activation of diacylglycerol (DAG) and calcium-dependent conventional PKC (cPKC), which attenuates IRS–PI3K–PDK1–AKT signaling pathway. In addition, stimulation of the cGMP–NOS pathway also assaults this signaling pathway by nitrosylation of IRS. Thus, PST-mediated suppression of this pathway allows forkhead box protein O1 (FoxO1) and sterol regulatory element-binding transcription factor 1c (SREBP1c) to stimulate expression of gluconeogenic genes, phosphoenolpyruvate carboxykinase 1 (*Pck1*) (also known as *Pepck*) and glucose-6-phosphatase (*G6pc*) (also known as *G6Pase*), and thus prevent insulin action. Under control conditions, insulin would have activated this signaling pathway, causing phosphorylation of FoxO1 (promoting its exclusion from the nucleus) and preventing processing of SREBP1 proprotein to SREBP1c with consequent inhibition of expression of gluconeogenic genes and gluconeogenesis. **(C)** Catestatin (CST) stimulation of lipolysis in adipocytes. Activation of α2-adrenergic receptor (α2-AR) inhibits β1/2-AR-induced lipolysis in a dominant way in obesity. CST enhances lipolysis by inhibiting α2-AR, which promotes β1/2-AR action and the consequent downstream signaling. Hormone-sensitive lipase (HSL) is an intracellular, neutral lipase that has broad substrate specificity, catalyzing the hydrolysis of triacylglycerol (TAG), diacylglycerol (DAG), monoacylglycerol (MAG), and cholesteryl esters. Its activity against DAG is about 10- and 5-fold higher than its activity against TAG and MAG, respectively, whereas its activity against cholesteryl esters is about twice its activity toward TAG. The hydrolytic activity of HSL against TAG and cholesteryl esters, but not against DAG, is stimulated by phosphorylation mediated primarily by PKA (84). AC, adenylyl cyclase; FFA, free fatty acids; MGL, monoacylglycerol lipase; PKA, protein kinase A. **(D)** Summary of PST and CST actions.

PST, a C-terminally glycine-amidated 49-mer peptide, was identified in 1986 as a potent inhibitor of glucose-stimulated insulin secretion (GSIS) (32). Two molecular forms were detected in human plasma: a 52 amino acid form (CgA_250–301_) and a larger form with a molecular weight of 15–21 kDa (65). Although the PST sequence is well conserved in mammals, showing 41.5% homology between humans and the Tasmanian devil, no homology could be detected in submammalian vertebrates (Figure 1B) (66–68). PST inhibits GSIS *in vivo* in mice, rats, dogs, and pigs, as well as *in vitro* from isolated rat islets (69). In the perfused rat pancreas, PST inhibits unstimulated and stimulated insulin secretion (70–73). In PST-deficient *Chga*-KO mice, GSIS was ~1.7-fold higher at 7 and 15 min after administration of glucose, confirming the inhibitory role of PST in GSIS (60). In addition, PST inhibits glucagon secretion induced by low glucose (74) but had no effect on somatostatin secretion (75). In addition to inhibition of GSIS, PST inhibits insulin-stimulated glucose transport in primary rat and mouse adipocytes (60, 76, 77), differentiated 3T3-L1 adipocytes (68, 78), and primary hepatocytes (60). PST also increases nitric oxide (NO) levels in HTC rat hepatoma cells (79), L6 myotubes (68), and in livers of *Chga*-KO mice (60), showing that PST inhibits insulin action. Since NO inhibits GSIS (80) and PST increases NO production (60, 68, 79), we believe that PST likely inhibits GSIS through activation of the NO pathway (Figure 2A).

## PST Regulates Hepatic Glucose Metabolism

PST treatment inhibits insulin-stimulated glycogen synthesis in primary hepatocytes (81) and activates glycogenolysis in the rat liver, implicating a direct anti-insulin effect on liver metabolism (82, 83). PST-deficient *Chga*-KO mice show greater suppression of hepatic glucose production (HGP) compared to wild-type (WT) mice during insulin clamp studies (60). Decreased glucose production in *Chga*-KO mice was also supported by decreased glucose production during pyruvate tolerance tests and decreased mRNA transcript levels of the gluconeogenic genes, such as the phosphoenolpyruvate carboxykinase 1 and glucose-6-phosphatase (*G6pc*), compared to WT mice that were restored to WT levels after supplementation of PST to *Chga*-KO mice (60). PST activates gluconeogenesis by decreasing phosphorylation of insulin receptor substrate 2 at tyrosine residues through activation of conventional PKC and increases production of NO with subsequent attenuated phosphorylation of protein kinase B (AKT), forkhead box protein O1, and reduced matured sterol regulatory element-binding transcription factor 1c (SREBP1c) (Figure 2B) (60). These findings are consistent with the anti-insulin action of PST.

## PST Influences Lipid Metabolism

In addition to glucose metabolism, PST also modulates lipid metabolism. PST decreases insulin-stimulated synthesis of lipids in rat adipocytes (85), which is consistent with the PST-dependent increased expression of hepatic lipogenic genes in *Chga*-KO mice, including *Srebp1c*, peroxisome proliferator-activated receptor-gamma, and glycerol-3-phosphate acyltransferase (*Gpat*) (60). PST also stimulates release of glycerol and free fatty acids from rat adipocytes, which is completely inhibited by insulin (85). In humans, PST augments free fatty acid efflux into the circulation, resulting in an overall spillover of ~4.5-fold, which is consistent with the reported lipolytic action of PST (85), confirming the anti-insulin effects of PST.

## PST Promotes Inflammation and Insulin Resistance

Since PST inhibits the action of insulin on glucose and lipid metabolism, one would expect improved insulin sensitivity in PST-deficient mice. Indeed, *Chga*-KO mice show improved hepatic insulin sensitivity as assessed by insulin tolerance tests (ITTs) showing increased hypoglycemia, and insulin clamp studies showing increased suppression of HGP. Improved hepatic insulin sensitivity was abolished when *Chga*-KO mice were treated with PST, implicating a positive correlation between PST and the development of insulin resistance (60). Similarly, type 2 diabetes mellitus (T2DM) patients show a substantial increase in plasma PST levels (~3.7-fold) (77). Gestational diabetic subjects and patients with non-insulin-dependent diabetes mellitus also show increased plasma PST levels (86, 87).

Feeding mice a high fat diet (HFD) creates obesity, leading to hyperinsulinemia and inflammation (88–92). ITT studies revealed that HFD-fed *Chga*-KO mice displayed improved insulin sensitivity compared to WT mice, demonstrating the importance of PST in the development of IR (64). This was reinforced by hyperinsulinemic–euglycemic clamp studies, where *Chga*-KO-HFD mice displayed increased glucose infusion rates, higher insulin-stimulated glucose disposal rates (IS-GDRs), and suppressed HGP. Recent studies implicate dissociation between obesity and insulin resistance as long as the inflammation is suppressed (64). The presence of supraphysiological levels of PST can reconnect obesity with insulin resistance by introducing inflammation. In the absence of PST, animals are insulin sensitive despite obesity. This is reminiscent of rosiglitazone-treated WT-HFD mice, which are insulin sensitive but obese (93–95).

The hallmarks of insulin resistance in HFD mice are obesity, hyperinsulinemia, and increased inflammation (88–92). Suppression of inflammation in HFD mice can improve insulin sensitivity (93–95). Therefore, the resistance to diet-induced insulin resistance in *Chga*-KO mice may reflect less inflammation in *Chga*-KO mice even after HFD feeding. PST treatment caused increased expression of the pro-inflammatory genes interleukin 1-beta, tumor necrosis factor alpha (*Tnfa*), interleukin 6 (*IL6*), chemokine C–C motif ligand 2 (*Ccl2*), and nitric oxide synthase 2a. Whereas expression of anti-inflammatory genes such as arginase 1 (*Arg1*), interleukin 10 (*IL10*), and C-type lectin domain family member 10a (*Clec10a*) in adipose tissues was higher in *Chga*-KO-HFD mice than WT controls, PST treatment significantly reduced the expression of *Arg1* and *IL10*. Consistent with gene expression data, the plasma levels of IL12p70, Ifng, and chemokine C–C motif ligand 3-like 1 (Ccl3l1), IL6, and chemokine C–X–C motif ligand 1 (Cxcl1) showed significantly decreased levels in *Chga*-KO-HFD versus WT-HFD plasma. PST treatment of *Chga*-KO-HFD mice raised plasma levels of IL12p70 and Ccl2, but had no effect on other proteins measured. PST also exerted direct effects on peritoneal macrophage cultures obtained from WT and *Chga*-KO mice. CgA-deficient peritoneal macrophages demonstrated attenuated response to LPS in the expression of pro-inflammatory cytokines as well as decreased chemotaxis in response to cytokines (64). PST treatment increased the expression of *Tnfa* and *Ccl2* in *Chga*-KO macrophages (64). Thus, it appears that PST acts as a pro-inflammatory peptide but its loss is likely only partially responsible for the improved inflammation seen in *Chga*-KO mice (Figure 2D).

Although clamp studies with *Chga*-KO mice fed normal chow diet (NCD) indicated decreased glucose disposal, meaning muscle insulin resistance (60), surprisingly, reduced muscle insulin sensitivity in lean *Chga*-KO mice was reversed by HFD feeding as demonstrated by improved IS-GDR in muscle of HFD-fed *Chga*-KO mice. Can feeding a high amount of lipids to CgA-deficient mice regenerate cells and repair muscle dysfunction? What kind of lipid could that be? These unorthodox results on the regulation of muscle insulin sensitivity by a CgA-derived protein need further investigation. In this regard, one provocative speculation may deserve some investigation. HFD-induced ceramide and sphingolipids were implicated in the mobilization and differentiation of bone marrow-derived stem/progenitor cells, which are involved in the repair of tissues in ischemic heart disease (96). More specifically, sphingosine-1-phosphate (S1P) acts as a trophic factor for skeletal muscle cell regeneration (97). Sphingolipids are important structural components of cell membranes and are derived from ceramide. Ceramide production is increased in obesity and after HFD feeding (98, 99). Ceramide can be deacylated to sphingosine, which is then phosphorylated by sphingosine kinases to yield S1P. Since this improvement in muscle insulin sensitivity by HFD happened in *Chga*-KO mice, not in WT-DIO mice, absence of CgA protein or peptides triggered this unusual phenomenon. Therefore, it will be very important to investigate the roles of these dietary lipids in muscle repair and the functional relationship of these lipids with the CgA protein and CgA-derived peptides. Alternatively, it is also possible that the absence of CgA protein and its derivatives stimulated release of some myokines in response to dietary lipids, which would otherwise remain suppressed in WT-DIO mice. This response to HFD in *Chga*-KO mice could be muscle specific because muscle expresses CgA (100), and liver and adipose tissue do not (3, 46). Effects of CgA deficiency on liver and adipose tissue may be more systemic in nature, a part of which is carried out by CgA-deficient macrophages (64).

## PST Promotes Endoplasmic Reticulum (ER) Stress by Attenuating Expression of Grp78

The accumulation of unfolded and misfolded proteins in the ER lumen, termed ER stress, leads to activation of signaling pathways to counteract defects in protein folding (101–106). This unfolded protein response (UPR) increases repair activities, reduces global protein synthesis, and activates ER-associated protein degradation. However, if ER stress becomes chronic and UPR cannot cope with the repair demands, protein-folding homeostasis breaks down, leading to activation of apoptotic pathways (103, 107, 108). Thus, ER stress and the UPR play important roles in the pathogenesis of multiple human metabolic diseases including insulin resistance, diabetes, obesity, non-alcoholic fatty liver disease, and atherosclerosis (109, 110). The immunoglobulin binding protein (BiP) [also called glucose-regulated protein 78 (Grp78)], is an ER chaperone that is required for protein folding. BiP/Grp78 is a peptide-stimulated ATPase of the Hsp70 family that prevents protein aggregation by stabilizing intermediates in the protein-folding process.

Using ligand affinity chromatography with biotinylated human PST (hCgA_273–301_-amide) as “bait” on a murine liver homogenate (as “prey”), we found that PST interacts in a pH-dependent fashion with Grp78 (78). Whereas NCD-fed *Chga*-KO livers show increased expression of Grp78, PST caused dose-dependent inhibition of Grp78 ATPase activity and inhibited increased expression of Grp78 during UPR activation (by tunicamycin) in hepatocytes (78). In hepatocytes, PST increased expression of *G6pc*. These results indicate that a major hepatic target of PST is the adaptive UPR chaperone Grp78 and that ATPase activity associated with Grp78 is involved in the suppression of glucose production by attenuating *G6pc* expression (78). Grp78s ATPase activity is required to suppress expression of *G6pc*; ER stress and suppression of glucose utilization appear to augment *Grp78* expression (111). Although it is not clear how circulating PST might contact the ER luminal protein Grp78 to modulate ER and insulin action, it has been reported that Grp78 translocates to the cell surface under some pathological conditions (112, 113).

## Modulation of Metabolism by Naturally Occurring Variants of PST

Single-nucleotide polymorphism analysis of PST, both *in vivo* and *in vitro*, showed greater inhibition of insulin-stimulated glucose uptake by **Gly**297Ser variants followed by the **Glu**287Arg variants compared to WT-PST (77). The *in vitro* studies also revealed increased expression of gluconeogenic genes by PST variants as compared to WT-PST, with comparable potencies by **Glu**287Arg and **Gly**297Ser variants (68). The **Gly**297Ser subjects displayed markedly elevated plasma glucose and cholesterol compared to the **Gly**297**Gly** individuals. Interestingly, whereas the variants of PST in the C-terminal half of the molecule at 287 (Glu287Arg) and at 297 (Gly297Ser) enhance anti-insulin effects and elevate plasma glucose by inhibition of glucose uptake and stimulation of gluconeogenic effects, experimental deletion of the three N-terminal amino acids Pro–Glu–Gly on human WT-PST demonstrated the opposite effects by reducing plasma glucose level and hepatic gluconeogenesis in a rodent model of obesity (64). Therefore, finding variants in the N-terminal end of PST among the human population may lead to discovery of an allele which would confer protection against insulin resistance and can be used as an insulin-sensitizing peptide such as a N-terminal variant of PST (lacking three amino acids from the N-terminal end) called PSTv1 (64).

## Regulation of Insulin Sensitivity by the PST Antagonist PSTv1

The elevated levels of plasma PST observed in T2DM patients (77) implied that preventing PST action might serve a therapeutic purpose of controlling insulin resistance and diabetes. To demonstrate a direct *in vivo* role of PST in the regulation of insulin sensitivity, WT-HFD mice were injected with the PST variant, PSTv1, which is a competitive antagonist of native PST. PSTv1 lacks the first three N-terminal residues of native PST and blocks PST-mediated inhibition of glucose uptake and leptin secretion in 3T3-L1 preadipocytes. As predicted, chronic PSTv1 treatment lowered fasting plasma glucose levels in WT-HFD mice and improved glucose tolerance and insulin sensitivity (64). These results suggested that in WT-HFD mice, where the level of PST is high, PSTv1 administration competes with the native PST and phenocopies *Chga*-KO mice. This demonstrates the potential of PST as a therapeutic target for treatment of insulin resistance and diabetes.

## CST Decreases Hypertension and Obesity

Hypertensive patients show elevated levels of plasma CgA but decreased plasma CST (114, 115). Low plasma CST predicts augmented pressor responses to environmental stimuli (114). In rats, CST reduces blood pressure responses to activation of sympathetic outflow by electrical stimulation (116). This vasodepressor effect of CST was mediated by massive release of histamine with subsequent vasodilation by histamine-induced production of NO. CST is a potent endogenous inhibitor of catecholamine secretion (44–47, 117–120) and catecholamine-mediated hypertension (48, 121). *Chga*-KO mice showed hyperadrenergic and hypertensive phenotypes that were normalized by intraperitoneal administration of CST (48). CSTs hypotensive effect was also documented in a polygenic model of high blood pressure mice (121). Other studies showed that CST also provides cardioprotection by inhibiting the opening of the mitochondrial permeability transition pore and stimulating the reperfusion injury salvage kinase pathway (122–127).

Catestatin-deficient *Chga*-KO mice are obese on an NCD (48). Chronic CST administration to *Chga*-KO mice reduced epididymal fat pad size to WT level (~25% reduction with respect to body weight of *Chga*-KO mice) (55). CST decreased plasma triglyceride levels in *Chga*-KO mice by increasing lipolysis (increased plasma glycerol and non-esterified fatty acids) through inhibition of α2-adrenergic receptor (α2-AR) (Figure 2C) (55). While inhibition of α2-AR by CST indirectly facilitates β-AR mediated lipolysis, CST can also have direct effect on ATGL (adipose triacylglycerol lipase) and HSL (hormone sensitive lipase) via activation of AMPK (128) as it has been demonstrated that activation of AMPK promote lipolysis in adipose tissue through ATGL and HSL. CST-treated *Chga*-KO mice show increased palmitate oxidation but decreased incorporation into lipids, which indicates that CST inhibits expansion of adipose tissue but promotes fatty acid uptake in the liver for oxidation. CST induced expression of several fatty acid oxidation genes including carnitine palmitoyltransferase 1a, peroxisome proliferator-activated receptor-a, acyl-CoA oxidase 1, and uncoupling protein 2, supporting increased fatty acid oxidation in the liver. In addition, CST increased expression of the fatty acid transporter gene *Cd36* and the lipogenic gene glycerol-3-phosphate acyltransferase 4 (*Gpat4*), indicating that CST stimulates fatty acid incorporation into triglycerides but not *de novo* lipogenesis. Overall, CST promoted lipid flux from the adipose tissue toward the liver for beta-oxidation (55). These obesity-reducing effects of CST are mediated by inhibition of α2-AR signaling and enhancement of leptin receptor signaling. In contrast to the negative metabolic effects of PST, CST has beneficial effects that could be utilized in therapeutic treatment of hypertension and obesity.

## Conclusion and Future Perspectives

Chromogranin A is one of the few protein molecules, which can be processed into both negative and positive regulators such as PST and CST for fine-tuning and maintaining metabolic homeostasis. With respect to the pathway of lipid disposal, studies on the direct effect of CST, through activation of AMPK, on lipolytic activities of ATGL and HSL may generate exciting information. Although the metabolic effects of PST and CST have been well investigated, how they transmit signals into cells remains to be determined. Are there specific receptors for these peptides? Alternatively, can they opportunistically bind to some non-specific BiPs on the cell surface and get endocytosed? In some cells such as neutrophils, CST has been shown to be permeable (53). With respect to PST, its binding to Grp78 may occur opportunistically on the cell surface when Grp78, usually a luminal protein, translocates to the cell surface, which occurs under some pathological conditions (112, 113). Whether such interaction happens or not should be a matter of future investigation. If that happens, Grp78 would be able to carry PST to the luminal compartment and initiate a reaction with a small G-protein binding molecule leading to a cascade described in Figure 2B. In addition, although PST has been established as an anti-insulin peptide, the mechanisms underlying PST-dependent regulation of insulin secretion are poorly understood. Other CgA-derived pro-insulin peptides may also exist and need to be further investigated. These efforts, as well as generation of PST antagonists, may lead to development of powerful therapeutic treatments for insulin resistance and diabetes. Beyond PST and CST, additional studies should shed light on the role of other CgA-derived peptides in metabolism, with implications for treatment of metabolic disease.

## Author Contributions

SM conceived the idea. GB and SM contributed equally to researching the data and writing of the manuscript.

## Conflict of Interest Statement

The authors declare that the research was conducted in the absence of any commercial or financial relationships that could be construed as a potential conflict of interest.
